# AMIC Cartilage Repair in a Professional Soccer Player

**DOI:** 10.1155/2012/364342

**Published:** 2012-09-17

**Authors:** S. Bark, H. Riepenhof, J. Gille

**Affiliations:** Klinik für Chirurgie des Stütz und Bewegungsapparates, Sektion für Unfallchirurgie, Universitätsklinikum Schleswig-Holstein, Campus Lübeck, Ratzeburger Allee 160, 23538 Lübeck, Germany

## Abstract

We report a case of a professional soccer player suffering from a traumatic cartilage lesion grade IV according to the Outerbridge classification at the femoral condyle treated with an enhanced microfracture technique (AMIC). Autologous Matrix-Induced Chondrogenesis (AMIC) is an innovative treatment for localized full-thickness cartilage defects combining the well-known microfracturing with collagen scaffold and fibrin glue. Because of the cartilage lesion (3 cm^2^), an AMIC procedure was performed followed by a rehabilitation program according to the protocols in the literature, (Steadman et al.; 2003). After 8 months of rehabilitation, the player returned to team training and after 10 months to competition. Altogether he returned to the same skill level for almost one year after the index operation. He is very satisfied with the clinical results after AMIC, which corresponds with the Lysholm score of 90 points at 12 months.

## 1. Introduction

Articular cartilage defects are one of the most common causes of permanent disability in athletes. Excessive stress on a joint with an articular cartilage defect may accelerate further degenerative changes and predispose the athlete to a higher risk of osteoarthritis. Athletes require an articulating cartilage surface that can withstand the high mechanical joint stresses generated during their specific sports activity. Treatment of an athlete with articular cartilage damage is consequently a significant challenge to healthcare professionals. We report a case of a professional soccer player suffering from a traumatic cartilage lesion treated with an enhanced microfracture technique (AMIC).

## 2. Case Report

**Figure 1:**
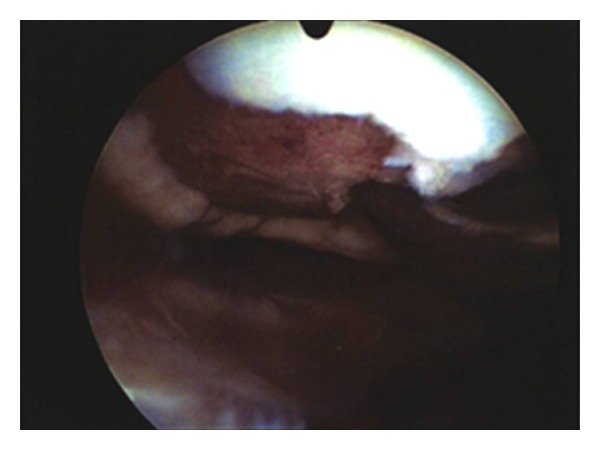
Arthroscopic photograph of the femoral condyle showing an extensive cartilage defect rated as grade IV according to the Outerbridge classification. The fresh blood clot in the defect area pinpoints to a traumatic origin of the lesion.

A 28-year-old professional soccer player suffered an anterior cruciate ligament tear after a tackling in a soccer game. He underwent a routine anterior cruciate ligament reconstruction with use of quadruple hamstring autograft and bioabsorbable fixation of the implants. He recovered well and returned to competition after 6 months. Another 2 month later he injured his knee hitting the ground with the lateral side of the knee. He referred immediately discomfort but no apparent effusion and he continued to play on. After one week, the discomfort progressed despite physiotherapy. An orthopaedic assessment was made for persistent pain located at the lateral aspect of the knee. At that time the treating surgeon performed a diagnostic arthroscopy and it was confirmed that the patient had a cartilage lesion grade IV according to the Outerbridge classification at the femoral condyle (Figure 1). He was referred to the authors for further treatment. At the time of surgery, a complete examination under anaesthesia showed normal knee laxity under manual laxity tests with no signs of rotator instability. Because of the cartilage lesion (3 cm^2^) an AMIC procedure was performed followed by a rehabilitation program according to the protocols in the literature [1].

## 3. Results

**Figure 2:**
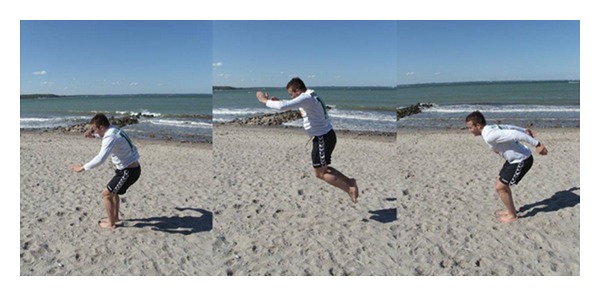
To demonstrate the functional outcome, a squat jump was documented.

After 8 months of rehabilitation following a standardized protocol, the player returned to team training and after 10 months to competition. Altogether he returned to the same skill level for almost one year after the index operation. He is very satisfied with the clinical results after AMIC, which corresponds with the Lysholm score of 90 points at 12 months (Figure 2). To date he is working in a marketing department of a sports club but still plays recreational soccer without limitations.

## 4. Discussion

Soccer remains the most popular sport in the world, with more than 22 million participants annually [2]. The rapid deceleration pivoting actions combined with the kicking motion and contact with other players leave knees extremely vulnerable to injury. Overall, knee injuries reportedly account for approximately 20% of the injuries in soccer players [3]. In a recent study an increasing frequency of chondral injuries in collegiate, professional, and world-class soccer players has been described [4]. Our clinical experience has demonstrated that soccer injuries can involve a fracture of the surface and a separation of the uncalcified layer from the calcified cartilage. 

Because of the significant mechanical stress on the articular surface of the knee when playing soccer, studying cartilage repair in this particular athletic cohort offers a valid option to examine whether surgical treatment provides a reliable repair of articular cartilage lesions under high demands. Besides the frequently used validated functional outcome scores, the ability to return to demanding athletic activity presents a very important parameter for functional outcome evaluation [5].

Focal articular cartilage defects have been recognised to be progressive, leading to deterioration, therefore, early diagnosis and treatment are recommended prior to the development of more advanced osteoarthritis [6]. Treatment of cartilage defects is surgical, but opinions differ as to its modalities. Besides autologous chondrocyte implantation [7], other techniques for the repair of articular cartilage defects have been evaluated in athletes, such as microfracture [5] and osteochondral mosaicplasty [8].

Using the microfracture technique, Blevins et al. demonstrated that 77% of their elite athletes were able to return competition [9]. Similarly, Steadman et al. reported a return rate of 76% in National Football League players after microfracture [1]. 

Returning to sports and exercise activity is one of the main reasons given for individuals electing to undergo cartilage repair surgery. A return to preinjury performance levels is not guaranteed, and for some athletes articular cartilage injuries may be career-ending. Not all athletes who are physically ready and able to return to their sport actually do so. In a study by Mithöefer and colleagues, 33% of soccer players returned to football after autologous chondrocyte implantation [7]. The return rate was significantly higher in high-level competitive athletes than in recreational-level players. Of the returning players, 80% returned to the same skill level and 87% maintained it for an average of 52 months postoperatively. As shown in our case, it should be recognized that cartilage repair rehabilitation is a long process. This is in accordance with the literature, reporting a time to return to sports of 7 to 18 month after cartilage repair procedures [10]. Behind this background it should be kept in mind that during this long rehabilitation period also psychosocial factors have an impact on return-to-sport outcomes [11]. 

In summary, the results of the presented case with the use of the AMIC in a professional soccer player are encouraging. After rehabilitation he returned to sport at the same skill level as prior to the accident. In general, long-term evaluation will help to determine whether the restoration of articular cartilage lesions in the knee in soccer players can effectively reduce the high incidence of osteoarthritis in this population. 

The limitation of this case report has to do with the extent to which the findings can be generalized beyond the case studied.
